# Virus Reduction Neutralization Test: A Single-Cell Imaging High-Throughput Virus Neutralization Assay for Dengue

**DOI:** 10.4269/ajtmh.17-0948

**Published:** 2018-10-22

**Authors:** Melissa C. Whiteman, Leah Bogardus, Danila G. Giacone, Leonard J. Rubinstein, Joseph M. Antonello, Dengyun Sun, Sarah Daijogo, Kevin B. Gurney

**Affiliations:** 1 PPDM Bioanalyitical Regulated Immunogenicity and Molecular, Merck and Co., Inc., Kenilworth, New Jersey;; 2 Biologics Analytical Sciences, Merck and Co., Inc., Kenilworth, New Jersey;; 3 PPDM Bioanalyitical Regulated Immunogenicity and Molecular, Merck and Co., Inc., West Point, Pennsylvania;; 4 Biometrics Research, Merck and Co., Inc., West Point, Pennsylvania;; 5 Q^2^ Solutions Vaccines, San Juan Capistrano, California

## Abstract

Vaccine immunogenicity and clinical efficacy are often assessed by the measure of serum-neutralizing antibodies. The present gold standard for detecting neutralizing antibodies against many viruses, including dengue, is the plaque/focus reduction neutralization test (P/FRNT). The FRNT is a cell-based assay that inherits high variability, resulting in poor precision and has lengthy turnaround times. The virus reduction neutralization test (VRNT) is a high-throughput alternative to the standard low-throughput and laborious FRNT. The VRNT is similar to FRNT using unaltered wild-type virus and immunostaining, yet uses imaging cytometry to count virus-infected cells 1 day post-infection, reducing assay time and increasing overall throughput 15-fold. In addition, the VRNT has lowered variability relative to FRNT, which may be explained in part by the observation that foci overlap alters foci count and titer over time, in the FRNT. The ability to count one infected cell, rather than waiting for overlapping foci to form, ensures accuracy and contributes to the precision (7–25% coefficient of variation) and sensitivity of the VRNT. Results from 81 clinical samples tested in the VRNT and FRNT show a clear positive relationship. During sample testing, a 96-well plate edge effect was noted and the elimination of this edge effect was achieved by a simple plate seeding technique. The VRNT is an improvement to the current neutralization assays for its shortened assay time, increased precision and throughput, and an alternative to the P/FRNT.

## Introduction

Dengue (DEN) is a mosquito-borne positive-sense RNA virus of the family *Flaviviridae*, genus *Flavivirus* with four distinct serotypes (DEN 1–4). Dengue affects more than 40% of the world’s population and is the leading cause of illness and death in young children in the subtropical and tropical areas of the world.^1^ Subsequent infection with a DEN virus strain is a risk factor for severe disease and is associated with 90% of DEN hemorrhagic fever (DHF) cases, whereas the remaining 10% that present DHF after primary infection are predominately children within the 1st year of life.^2^ Hyperendemic areas circulate all four serotypes; thus, a vaccine for DEN should be tetravalent, provide a balanced immune response,^3^ and protect young children. One vaccine, Sanofi’s Dengvaxia^®^ (CYD-TDV), is presently licensed in several endemic countries for individuals aged 9–45 years, with an age-dependent vaccine efficacy of 45–66%.^4,5^ This vaccine is contraindicated in young children (< 9 years) because of the lack of efficacy in naive subjects and supported by increased hospitalizations in vaccinated 2–5 year olds in phase III trials. The WHO set a goal of reducing DEN disease by 25% and death by 50% by 2020 worldwide,^6^ therefore, there is a great need for an improved DEN vaccine particularly one which is efficacious in younger children.

The ability of a vaccine to elicit an immune response is essential to vaccine development and is important in determining clinical efficacy. A measure of the serum antibody response is a preferred method for assessing immunogenicity. Specifically, a functional neutralizing antibody test is a serological assay often performed to detect the amount of antibody that can effectively neutralize the virus, preventing infectivity in vitro. The current gold standard for many viruses, including DEN virus, is the plaque reduction neutralization test (PRNT), or similar focus reduction neutralization test (FRNT) which uses immunostaining to visualize “plaques” referred to as foci. The PRNT and FRNT are cell-based assays associated with high variability. In addition, some viruses are slow growing and may take up to 5 days to show infection via plaque or foci formation and with cell seeding and staining extending the duration of the assay up to 7 days in total resulting in lengthy turnaround times. The 24-well plate format has been used for DEN clinical sample FRNT testing,^7,8^ which can accommodate just one duplicate sample per plate for a 2-fold dilution scheme. The present DEN vaccine strategies are tetravalent,^9^ thereby, increasing the number of tests per sample 4-fold. For large studies, which include thousands of participants, the quantity of plates required becomes cumbersome, and testing plus analysis time is lengthy, therefore, randomized immunogenicity groups encompassing a subset of vaccinated subjects often replace complete sample testing.^10,11^ Thus, there is a great need to improve the present gold standard testing method to increase throughput and reduce assay time. Described herein is a higher throughput and rapid neutralization assay, the virus reduction neutralization test (VRNT).

## Materials and Methods

### Plate cell seeding.

Vero cells (ATCCCCL-81, Manassas, VA) were counted and measured for viability using a Vi-CELL^™^ XR (Beckman Coulter, Indianapolis, IN), before seeding to 96-well CellBIND^®^ black clear-bottom plates (Corning 3340, Corning, NY). A total of 30,000 cells were added per 150 µL 10% fetal bovine serum (FBS: Hyclone-SH30071.03HI, Omaha, NE), containing Minimal Essential Media (MEM: Gibco-11095 Gaithersburg, MD) supplemented with 1× nonessential amino acids (Gibco 11140), 1× L-glutamine (Gibco-25030), and 1× penicillin/streptomycin (Gibco-15140) per well. Plates were tapped lightly on each side to evenly distribute cells in the well, then placed in at 37°C with humidity and + 5% CO_2_ incubator overnight.

### Viruses and determination of virus dilution.

The viruses used for these experiments, WHO reference collection strains^12^ DEN1:West Pacific, DEN2:S16803, DEN3:CH53489, DEN4:TVP-360, were internally sourced. A large bank was generated by passaging the virus in Vero cells, centrifuging, aliquoting supernatant, and storing at −80°C until use. A virus titration was performed in 96-well plates using mock neutralization to determine the working dilution by serially diluting the virus 2-fold horizontally across the plate, combining 1:1 with 2% FBS MEM, incubation at 37°C for 30 minutes. Next 50 µL was transferred to the cell seeded plates (media removed before transfer) and incubating 30 minutes at room temperature (RT) was followed by incubation at 37°C overnight. Then cells were fixed and stained as described in the following paragraphs. The virus-infected cells were counted by image cytometry (as described in the following paragraphs). The development of the VRNT included setting an acceptance criteria of 500–8,000 object counts for the virus control, therefore, the dilution of the virus that was within this range was selected as the working dilution for VRNT.

### Neutralization and infection—assay day 1.

Two-fold serial dilutions of the sample were achieved by adding 20 µL sample to 180 µL media the first dilution in a 96-well plate (Costar-3879) and diluting 2-fold using a WellPro 3000 (ProGroup Instrument Corporation, Godfrey, IL) or manually pipetting (backup to automation and provides similar results—data not shown). Next, the previously determined working virus dilution was added to the sample and virus control wells. The serum sample dilutions and virus was mixed by pipetting or plate shaking at 300 rpm for 2 minutes and placed at 37°C for 30 minutes. The plates were then removed from the incubator and 50 µL was transferred to the cell seeded black 96-well plates (media removed before transfer). The virus was left to adsorb into the cells for 30–50 minutes at RT, followed by the addition of 150 µL of 2% FBS MEM to each well and the plates placed at 37°C overnight (22 ± 2 hours).

### Fixation and staining—assay day 2.

Approximately, 24 hours post-infection, the plates were removed from the 37°C incubator and left at RT for 10 minutes. The inoculum was removed, then cells washed with a single phosphate buffered saline (PBS) wash using a plate washer (BioTek Winooski, VT) or manually with a pipette before cooling the plates on ice and adding cold acetone. The plates were fixed at ‒20°C for 10 minutes, acetone removed, and the cells left at RT to dry. At this time, the cells were visually checked for holes in the monolayer. Blocking buffer (1% bovine serum albumin in PBS) was added to the well and cells were blocked for 1 hour at RT. After removing blocking buffer, primary antibody (rabbit anti-DEN antibody developed by Merck and Co., Inc., Kenilworth, NJ) diluted in blocking buffer at predetermined concentrations was added to the wells, and plates were incubated at 37°C without humidity or CO_2_ for 30 minutes. Primary antibody was removed and the wells were washed three times with PBS using the plate washer or manually. Alexa Fluor 488 secondary antibody (Invitrogen-A11070) was added at 1:1,000 diluted in blocking buffer, and plates were incubated at 37°C without humidity, for 30 minutes. The plates were again washed three times with PBS, residual PBS blotted before adding the final 200 µL of PBS.

### Object counting and analysis—assay day 2.

Plates were read using the SpectraMax^®^ i3X MiniMax^™^ 300 imaging cytometer (Molecular Devices, San Jose, CA) and corresponding SoftMax^®^ Pro (Molecular Devices) acquisition software version 6.5.1 or version 7.0 at a height of 14.2 mm, discrete object analysis with classification and region of interest off. Acquisition settings of wavelength 541, 0 µm focus, at a 45-ms exposure time with discrete object parameter at five minimum, 30 maximum and 125 greater than intensity. The MiniMax is capable of acquiring 12 sites (a 3 × 4 grid) per well, which includes the entire well plus areas outside the well. Four of the 12 possible sites per well (approximately 80% of the well) were acquired to reduce reading time per plate and to eliminate pseudo counts seen from auto fluorescence of the edge of the wells. Object count raw data acquired were exported as a text file and imported to a MS Excel workbook for processing.

Two, individually prepared, 10-point dilution series are tested for each sample. Each dilution from each series is tested in a single well, resulting in two wells per dilution. The dilution response curve for each serial dilution of a sample is fit in its entirety using the four-parameter logistic (4-PL) regression function. For all samples tested within a run, the lower asymptote of the 4-PL function (*D*) is set to the median of the 32 cell control (CC) counts and the upper asymptote of the 4-PL function (*A*) is set to the median of the 32 virus control (VC) counts. The values of the slope (*B*) and ED_50_ (*C*) (i.e., the dilution that results in 50% response) are estimated for each dilution series. The resulting estimates of *B* and *C* are the values that minimize the weighted residual sum of squares (i.e., provide the best fit to the data). A weighted fit (as opposed to an unweighted fit) is used to properly account for the proportionality between the mean and variance of the counts. The reported titer for a test sample is termed the VRNT_50_. This is the estimated dilution of the test sample that provides for a 50% reduction in response, where the response range is defined by the values of the CC and VC. The VRNT_50_ is the dilution at which the fitted curve has count (*A* + *D*)/2 and is given by the estimate of *C*.

Each assay run includes a negative quality control (NQC) (DEN-negative human serum) sample and an internal quality control (IQC) (DEN-positive human serum) sample, and each control sample is tested in duplicate on the control plate. The IQC and NQC were verified for positivity and negativity in both the VRNT and FRNT assays. A separate VRNT_50_ estimate is determined for each of the replicate dilution series. The validity of a run is based on the performance of the control plate and is assessed according to the following criteria: 1) The ratio of the median count of the VC to the median count of the CC must exceed 10; 2) the maximum count for the CC must be less than the average of the VC and CC median counts, termed the 50% cutoff count; 3) the minimum count for the VC must be greater than the 50% cutoff count; 4) the 4-PL regression function must have been successfully fit to each dilution series of the NQC and IQC; 5) each of the replicate VRNT_50_ estimates for the NQC must test negative (i.e., < 10); and 6) the replicate sample VRNT_50_ estimates for the IQC must be within 2-fold of one another. If one or more of the aforementioned criteria are not met, all plates within a run are considered invalid and all test samples within the run must be retested. In addition, the validity of each processed test sample is assessed according to the following criteria: 1) Each of the replicate dilution series must have been successfully fit using the 4-PL function; and 2) the replicate VRNT_50_ estimates for the sample must be within 2-fold of one another. If either criterion is not met, the test sample result is considered invalid and the test sample must be retested in a subsequent run.

### Virus reduction neutralization test assay development and optimization.

Virus reduction neutralization test assay development and optimization for the DEN1 and DEN2 viruses followed a design of experiments (DOEs) approach. Dengue 2 was the first virus developed in the VRNT. Because of the lack of clinical samples for the DEN2 DOE, a neutralizing antibody 4G2 (Millipore-MAB10216) was used as the sample. An eight-factor D-optimal split-plot design was created to identify the factors and the interaction of any two factors (2FI) that significantly affected the performance of the assay. The factors and levels of the factors investigated are shown in Table 1. The experiment required 14 96-well plates that were grouped into two groups each with seven plates to facilitate performing the experiment. Each row of each of the 14 96-well plates represented a single run condition identified by the Design–Expert software in creating the design. For each row the 4G2 neutralizing antibody was serially diluted by 10 2-fold dilutions starting at 1:40, followed by a “no virus” (negative) control and “no antibody” (virus) control. Each of the titration curves were then fitted with a weighted 4-PL regression. In addition, the precision (% coefficient of variation [CV]) at each of the points of the curve was calculated from the back-calibrated dilution.

**Table 1:** Design of experiment factors and levels investigated for DEN2

Factor	Whole plot or subplot	Level 1	Level 2	Level 3	Level 4
A. Plate incubation temperature	Whole plot	Ambient	37°C	N/A	N/A
B. Primary Ab incubation time and temperature	Whole plot	60 minutes/37°C	Overnight/4°C	N/A	N/A
C. Secondary Ab incubation time	Whole plot	30 minutes	90 minutes	N/A	N/A
D. Cell seeding density	Subplot	1 × 10^4^ cells	3 × 10^4^ cells	N/A	N/A
E. Multiplicity of infection	Subplot	0.01	0.1	N/A	N/A
F. Neutralization mix incubation-time/temp	Subplot	30 minutes/37°C	Overnight/4°C	60 minutes/37°C	60 minutes/ambient
G. Primary Ab concentration	Subplot	1 µg/mL	10 µg/mL	N/A	N/A
H. Secondary Ab dilution	Subplot	1:250	1:1,000	N/A	N/A

N/A = not applicable.

A positive human serum was used for the DEN1 DOE. The DEN1 DOE was a three-factor, two-level replicated design, which included four center point runs. Each run condition defined by the Design–Expert software was performed in duplicate pairs of rows (for example A and B) of each 96-well plate. Similar to the DEN2 method, the positive human serum was serially diluted across the plate as 10 2-fold serial dilutions starting at 1:10, followed by a negative control and virus control. The factors and levels are shown in Table 2. Each of the titration curves were then fitted with a weighted 4-PL regression.

**Table 2:** Design of experiment factors, levels and constant factors investigated for DEN1

	Level 1	Level 2
DOE factors		
A. Multiplicity of infection	0.01	0.1
B. Primary Ab concentration	1 µg/mL	2.5 µg/mL
C. Secondary Ab dilution	1:250	1:1,000
Constant factors		
D. Neutralization mix incubation temperature	Ambient	
E. Primary Ab incubation time and temperature	60 minutes/37°C	
F. Secondary Ab incubation time	60 minutes/37°C	
G. Cell seeding density	30,000 cells/well	
H. Neutralization time and temperature	30 minutes/37°C	

DOEs = design of experiments.

### Focus reduction neutralization test titers over 4 days.

Twenty-four well plates were seeded with 100,000 Vero cells per well and incubated at 37°C overnight. Neutralization was prepared in 96-well plates with a DEN-positive serum sample. Serial 2-fold dilutions starting at 1:10 and was combined 1:1 with 100 plaque-forming units (PFUs) virus (50 PFU final). One virus dilution was prepared for all plates. The neutralization plates were placed on the plate shaker for 2 minutes at 300 rpm to mix before placing at 37°C for 30 minutes to neutralize. One hundred microliters were added to 24-well cell plates and incubated at RT for 30 minutes before placing plates at 37°C for 1–4 days. Each day (days 1, 2, 3, and 4) plates were fixed with −20°C acetone for 15 minutes, then left to dry before blocking for 1 hour at RT. Primary antibody (rabbit anti-DEN custom) was added at 1–2.5 µg/mL (virus dependent) and incubated for 30 minutes at 37°C. The primary antibody was washed three times with PBS before adding 1:500 secondary antibody (KPL-4741516) and incubated at 37°C for 30 minutes. The secondary was washed three times with PBS before adding TrueBlue (KPL-207802) for 5 minutes, rinsing and drying plates.

### Virus reduction neutralization test specificity.

Three analysts tested the specificity of the VRNT using the DEN-specific monoclonal neutralizing antibodies (same as previously mentioned) as the test sample and yellow fever-positive human serum. Although DEN monospecific antisera was preferred to test for specificity of each of the four DEN assays, there were challenges to obtaining human sera positive to one DEN serotype, which was not available at the time of the study. In the test, each analyst tested 1) the DEN-specific target-neutralizing antibody (i.e., DEN1), 2) the nontarget neutralizing antibodies (i.e., DEN2, DEN3, DEN4), all four DEN neutralizing antibodies (DEN1, DEN2, DEN3, DEN4), three yellow fever–positive human serum samples (previously tested in the yellow fever FRNT assay, data not shown), the NQC and IQC. The specificity experiment was repeated for all four DEN viruses. The results for the three analysts were combined and the geometric mean titer result displayed.

### Edge effect.

Ninety-six–well CellBIND^®^ plates were seeded with 30,000 Vero cells per well. One plate was directly placed at 37°C, one plate was held at RT for 15 minutes before placing at 37°C, one plate held at RT for 30 minutes before placing at 37°C, and one plate was held for 45 minutes before placing at 37°C. One DEN-positive donor serum was added to each test sample position per plate and serially diluted 2-fold across the plate. One preparation of diluted virus for each serotype was used for all corresponding plates. Neutralization, infection, and staining proceeded as described previously. DRAQ5^™^ (Fisher-62252, Waltham, MA) was added with secondary antibody and counted along with virus-infected object counts.

### Virus reduction neutralization test/FRNT correlation.

Eighty-one samples from 21 subjects that were previously tested for DEN1–4 in the Q^2^ Solutions Vaccines FRNT were tested in the VRNT for comparison. The 81 samples were selected based on their DEN1–4 FRNT titers and were divided among baseline and postvaccination (placebo or active vaccine) time points. Serum was heat-inactivated before aliquoting and storing at −80°C until use. The VRNT was run as described previously.

For the purpose of estimating the titer ratio between assays, titers reported as < 10 were excluded from the quantitative titer comparisons. The functional relationship between assay methods was estimated using the linear statistical relationship (LSR) model.^13^ In comparing measurements between two assays, the LSR model, also referred to as an errors-in-variables model, is a regression model that recognizes that measurement error is present in both assays being compared. By contrast, standard regression models account for the presence of measurement error in just one of the two assays and regard the measures from the other assay as having been obtained exactly, without error. Failure to account for measurement error in both assays results in a biased (i.e. inaccurate) determination of the relationship between assays. In addition, the correlation between assay measures was estimated by the Pearson correlation coefficient and Lin’s coefficients for accuracy and concordance.^14^ Qualitative comparisons between assay methods were based on 2 × 2 cross-classification tables about the reciprocal of the minimum dilution of 1:10. From the 2 × 2 cross-classification tables, the agreement rate (proportion of double positive and double negative samples relative to the total number of samples) was reported. Cohen’s kappa coefficient, the rate of agreement beyond that which could be attributed to chance agreement, was also estimated.

## Results and Discussion

### Virus reduction neutralization test assay description.

**Figure 1.**
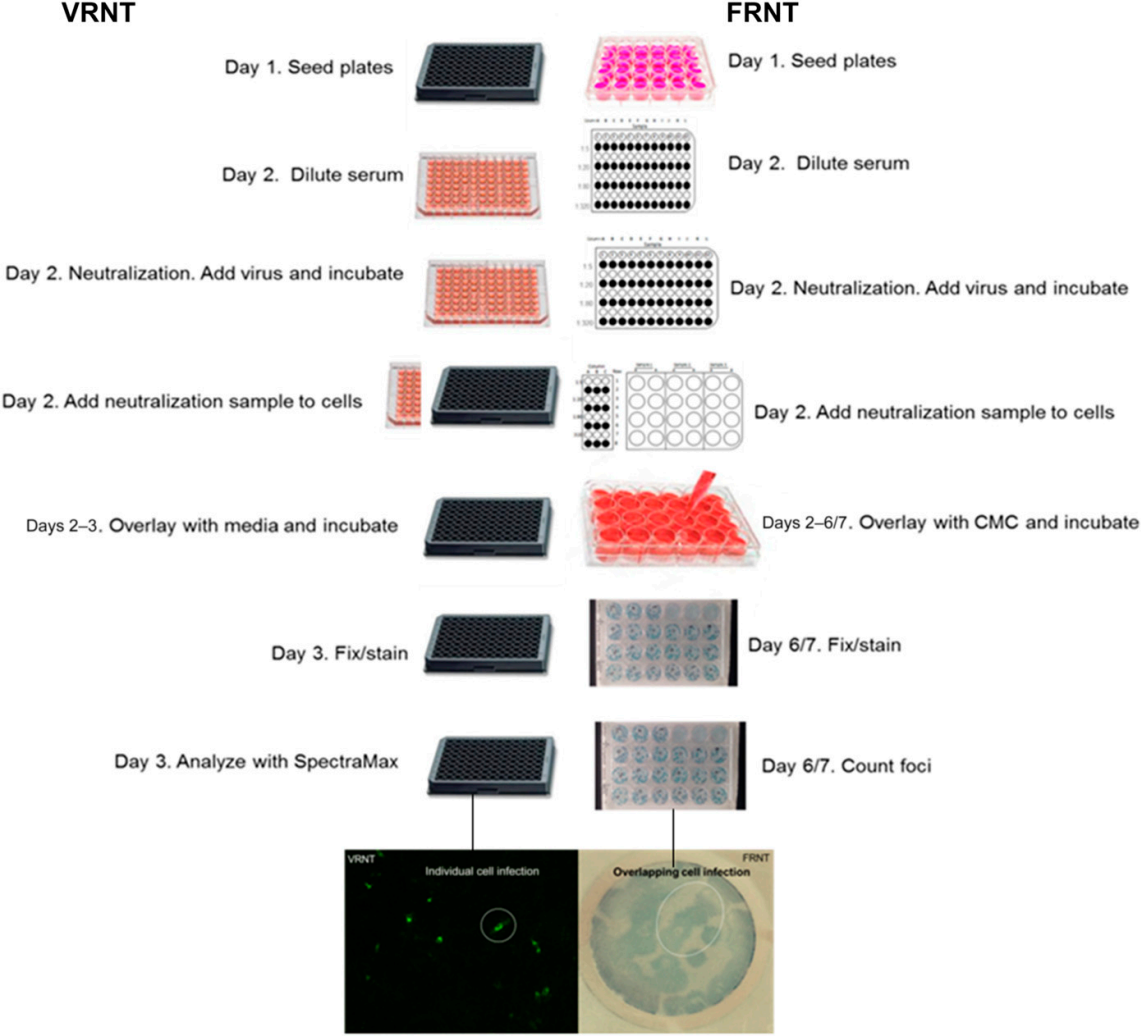
Side-by-side comparison of virus reduction neutralization test (VRNT) and focus reduction neutralization test (FRNT) methods. In this figure, day 1 is considered plate seeding rather than assay day 1. Differences include plate format (96-well for VRNT and 24-well for FRNT), infection time and secondary antibody and detection. Virus reduction neutralization test detection relies on fluorescent stained cells and cytometer counting; FRNT relies on peroxidase substrate and foci formation for manual foci counting. This figure appears in color at www.ajtmh.org.

The VRNT was developed to be an improvement to the PRNT and FRNT. The VRNT is similar to the FRNT in that the serum is serially diluted, then combined with a fixed amount of wild-type virus before adding this mixture onto cells. Like FRNT, 50% neutralizing titers are determined based on 50% reduction of the virus control and are calculated using the 4PL regression model. Different from FRNT, which counts immunostained foci several days postinfection, the VRNT reduces the total assay time to 2 days by imaging individually virus-infected cells 1 day postinfection using an imaging cytometer (Figure 1). The VRNT uses a 96-well format that increases samples (in duplicate) per plate up to 6-fold compared with the FRNT. The VRNT offers a number of advantages over the PRNT/FRNT, such as increasing throughput up to 15-fold, rapid turnaround, reduced sample volume requirements, and automation implementation, including automated serial dilution, plate washing, and the use of the SpectraMax^®^ i3x MiniMax^™^300 cytometer and SoftMax^®^ Pro microplate data acquisition and analysis software to count virus-infected cells, reducing manual labor.

### Virus reduction neutralization test assay development and optimization.

For the DEN2 analysis, the eight factors were assessed for their effect on 15 response variables. The response variables used were as follows: the object counts for the negative control, the values for each of the four parameters from the curve fitting (i.e., asymptotic maximum, Hill’s slope, inflection point, and asymptotic minimum), the calculated VRNT_50_ value, and the 10 estimates of precision (% CV) for each of the dilutions of the serially diluted 4G2 neutralizing antibody, for a total of 15 response variables. Each response variable was analyzed to determine which factors were significant in modeling that response. Only main effects or 2FI that were deemed significant (*P* < 0.05) in the analysis of variance (ANOVA) were included in the model. As expected, each response variable did not have the same factors or 2FI that were considered significant. A multiple-response optimization approach was taken instead of optimizing each response variable independently. The following constraints were used to find the overall best conditions for the assay. The negative control was targeted to be in the range of 0–20 object counts, the asymptotic maximum was targeted to be in the range of 300–5,000 object counts, the Hill’s slope had a target of one, and for each of the 10 dilution % CV, solutions were determined to minimize these values. The results of the simultaneous multiple response optimization is shown in Table 3. Confirmation experiments demonstrated that the levels for the factors chosen from the optimization indeed provided a reproducible assay with the desired outcome.

**Table 3:** Summary of optimal conditions for DEN2 design of experiments

Factor	Conclusion
A: Neutralization mixture plate incubation temperature	Ambient
B: Incubation time and temperature of primary antibody	60 minutes at 37°C
C: Secondary antibody incubation time	70 minutes
D: Cell seeding density	3 × 10^4^
E: Multiplicity of infection	0.01
F: Neutralization mixture time and temperature	30 minutes at 37°C
G: Concentration of primary antibody	1 µg/mL
H: Secondary antibody dilution	1:1,000

**Figure 2.**
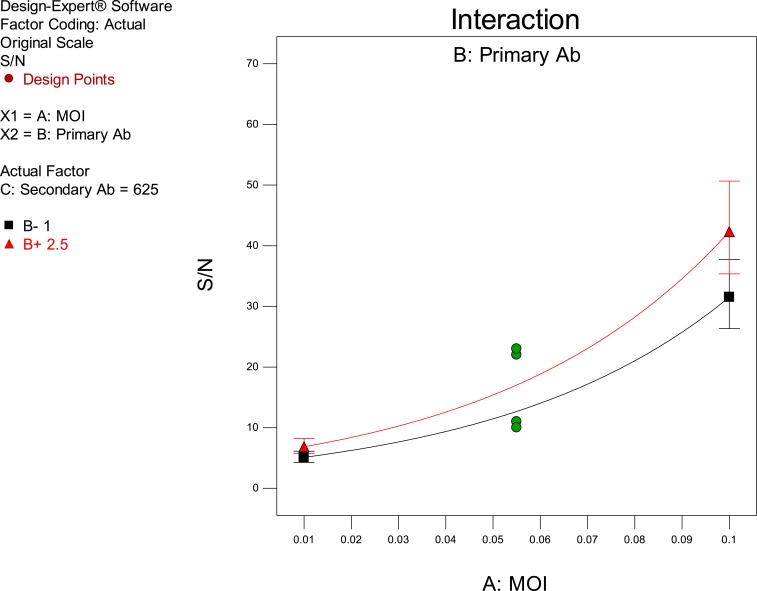
Interaction plot of multiplicity of infection (MOI) and primary antibody concentration on the signal-to-noise (S/N) Ratio. This figure appears in color at www.ajtmh.org.

For the DEN1 analysis, the three factors were assessed for their effect on two response variables. The response variables used were the Hill’s slope of the titration curve and the signal-to-noise (S/N) ratio of the virus control to the negative control. Only the main effects or 2FI that were deemed significant (*P* < 0.05) in the ANOVA were included in the model. Results from the analysis indicated that there were no main effects or interactions between factors that were significant for the Hill’s slope; thus, further analysis only focused on the S/N ratio. Only one factor was significant with a *P*-value less than 0.05 that was the multiplicity of infection (MOI). However, the *P*-value for the primary antibody concentration just passed the threshold of 0.05 and was therefore included in the model in the analysis of the S/N ratio. The results demonstrated that the S/N ratio in the experiment was maximized at the highest evaluated settings of MOI and concentration of primary antibody (Figure 2).

Because plaques or foci are not determined in the VRNT, MOI (PFU/cells) was replaced with a dilution of virus that was considered optimal and produced object counts greater than 500 for the “no antibody” control. Subsequent experiments evaluating the primary and secondary antibody incubation time of 60 minutes, demonstrated that this incubation time could be shortened to 30 minutes with no appreciable effect on the performance of the VRNT, noted by no change in the calculated VRNT_50_ titers. The information gathered from the development and optimization of the DEN1 and DEN2 assays was used in developing the VRNT for DEN3 and DEN4.

### Specificity of the VRNT assay.

The specificity of each of the four DEN VRNT assays was determined. In the experiment, DEN-specific monoclonal neutralizing antibodies were used to ensure no potential cross reactivity of the sample and positivity to only the input virus. Effects of having neutralizing antibody toward more than one DEN serotype in a test sample was also examined by combining the nontarget neutralizing antibodies in one test and a separate test with all four (DEN1, DEN2, DEN3, and DEN4) neutralizing antibodies. The results show specificity of each of the four DEN VRNT assays by positivity of the target sample and negativity when the three nontarget antibodies were tested. No inhibition or enhancement of neutralizing antibody titers was detected when the test sample that contained neutralizing antibodies toward all four DEN viruses was compared with the sample with the target only. The yellow fever virus–positive human serum samples also tested negative in each of the four DEN VRNT assays (Table 4).

**Table 4:** Specificity results for DEN1, DEN2, DEN3, DEN4 virus reduction neutralization test assays

	DEN1	DEN2	DEN3	DEN4
Target mAb	1,844	1,034	1,369	871
Non-target mAbs	< 10	< 10	< 10	< 10
All 4 mAbs	1,943	1,142	1,337	1,030
Negative quality control	< 10	< 10	< 10	< 10
Internal quality control	205	904	391	79
YF positive*	< 10	< 10	< 10	< 10

DEN = dengue; YF = yellow fever.

* Three individual samples were independently tested and all tested negative.

### Virus reduction neutralization test versus FRNT.

The VRNT platform demonstrates good precision. Over a 10-month period, a positive control serum sample was tested in the DEN2 assay across 54 runs performed across five analysts and two laboratories (Merck and Co., Inc., Kenilworth, NJ and Q^2^ Solutions Vaccines, San Juan Capistrano, CA) (Supplemental Figure 1). The overall CV of the VRNT_50_ titer across the 54 runs was 25%, with all 54 titers falling within 2-fold of the mean titer. This level of assay precision appears to be an improvement compared with other validated plaque reduction neutralization assays for DEN virus.^12^

**Figure 3.**
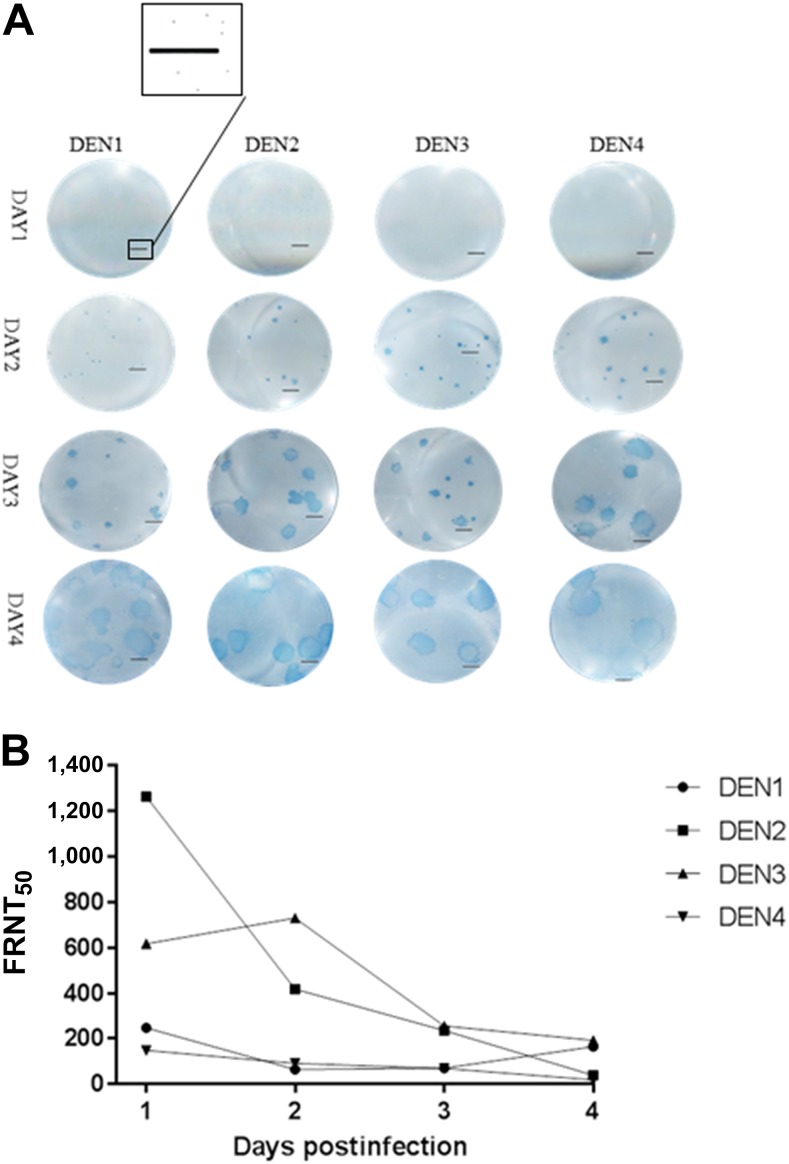
Focus reduction neutralization test over 4 days with DEN1–4 using a single positive donor serum sample. Foci development over 4 days reveals smaller foci can be in close proximity. Scale bar represents 2 mm. (**A**) FRNT_50_ titers over 4 days reveals a wide range of titers produced over 4 days (**B**). This figure appears in color at www.ajtmh.org.

Counting individually, virus-infected cells are hypothesized to contribute to the precision of the VRNT. Focus reduction neutralization test relies on counting foci manually, which often appear to overlap. Dengue FRNT assays range in infection time (incubation time) and virus input PFU between serotypes and laboratories. Because of these differences, FRNT titers can vary significantly between laboratories. In addition, highly variable results between laboratories present challenges to the interpretation of neutralization titers among vaccine trials.^15,16^ Laboratory-to-laboratory and day-to-day variations are well known for the FRNT, however, differences in foci formation could also contribute significantly to the inherent variability. To evaluate the impact of plaque size and potential overlap of foci on titer, FRNT_50_ titers were calculated for DEN1–4 over four consecutive days using 50 PFU of virus. At day 1, foci were not observed by eye, however, enlarged images revealed plaques, and by day 4, distinct foci were overlapping and difficult to count in many wells for all four viruses (Figure 3A). Reducing the days of infection reduced the foci size for each virus, and smaller foci appeared in close proximity indicating the potential for these smaller foci to fuse together to form larger foci over time (Figure 3A). The titers between days varied significantly (up to 30-fold) with highest titers when foci were the smallest (Figure 3B, Supplemental Table 1). When compared with VRNT titers for the same serum sample, the day 1 FRNT_50_ titers were within 1.5-fold of the corresponding VRNT_50_ titers for all four viruses (DEN1–4). This experiment indicates post–day 1 foci may consist of overlapping smaller foci, which would contribute to the inherent variability, as foci formation due to overlap could change. Conversely, VRNT only measures individual virus-infected cells and does not have the potential for overlapping or fused foci.

The DOE for DEN1 and DEN2 and subsequent experiments showed that the virus input range can be large (≥ 16-fold), and the VRNT_50_ titer remains consistent (within 1.5-fold). Thus, VRNT has additional advantages over the FRNT by eliminating the need to alter assay time to control for replication differences between viruses and the requirement to hit a narrow target (virus control foci count).

### Virus reduction neutralization test/FRNT correlation.

**Figure 4.**
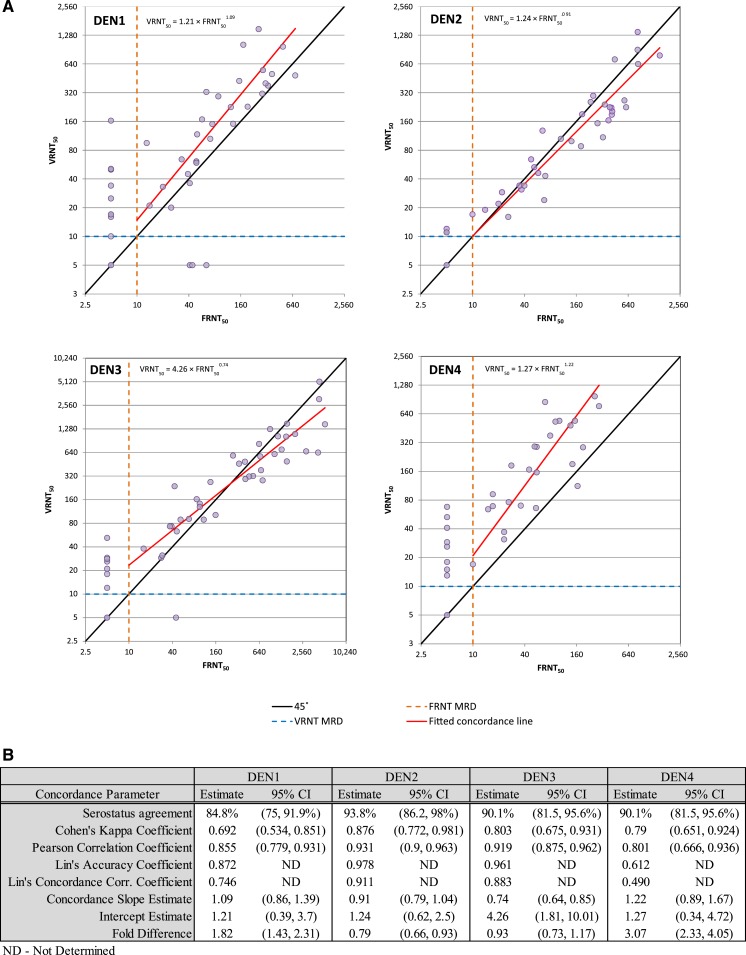
Virus reduction neutralization test (VRNT)/focus reduction neutralization test (FRNT) agreement. Virus reduction neutralization test_50_ and FRNT_50_ agreement plot for DEN1–4 (**A**). Measures of agreement between VRNT_50_ and FRNT_50_ assay procedures (**B**). This figure appears in color at www.ajtmh.org.

To assess the correlation between the FRNT and VRNT platforms, 81 DEN-vaccinated clinical serum samples that were previously tested against all four DEN serotypes in the FRNT were tested against the same four DEN serotypes in the VRNT (Supplemental Table 2). The results showed a clear positive association between the two assays for each of the four DEN serotypes (Figure 4A). Across the four serotypes, the fitted concordance slope ranged between 0.74 and 1.22, and the estimated Pearson correlation coefficient ranged between 0.80 and 0.93. For DEN3, the average fold difference in titer between VRNT_50_ and FRNT_50_ was not statistically significantly different from 1.0. For DEN2, VRNT_50_ titers were on average 1.27-fold lower (95% confidence interval (CI) = [1.08, 1.52-fold lower]) than FRNT_50_ titers, whereas for DEN1 and DEN4, VRNT_50_ titers were on average 1.82-fold higher (95% CI = [1.43, 2.31]) and 3.07-fold higher (95% CI = [2.33, 4.05]), respectively, compared with FRNT_50_ titers (Figure 4B). Dengue 4 virus produced the largest foci in the FRNT and had an 8-fold difference in titer between days 1 and 4 due to overlapping foci, making the assay more variable and less accurate, a possible reason for the 3-fold difference between VRNT_50_ and FRNT_50_ for this virus.

Both platforms (VRNT and FRNT) define a positive sample as NT_50_ ≥ 10 and negative sample as NT_50_ < 10 set by the minimum serum dilution. Across the four DEN types, of the 30 discordant results in expected positive samples (i.e., post DEN vaccination samples) 26 were positive in the VRNT (VRNT_50_ ≥ 10, range 10–163) and negative in the FRNT (FRNT_50_ < 10), and only four were negative in the VRNT (VRNT_50_ < 10) and positive in the FRNT (FRNT_50_ ≥ 10, range 41–64), suggesting higher sensitivity of the VRNT. In addition, of 84 expected negative test results (six placebo samples and 15 baseline samples tested across the four DEN types), 81 (96.4%) tested negative in the VRNT, indicating adequate specificity for the VRNT. For each of the three expected negative samples that tested positive in the VRNT, the VRNT_50_ titer was close to the minimum value (10), and the resulting positivity for each sample was attributed to the edge effect described in the following paragraphs. The performance of the VRNT assay on this dataset indicates that the VRNT can likely distinguish between true-positive and true-negative clinical samples.

### Edge effect elimination.

**Figure 5.**
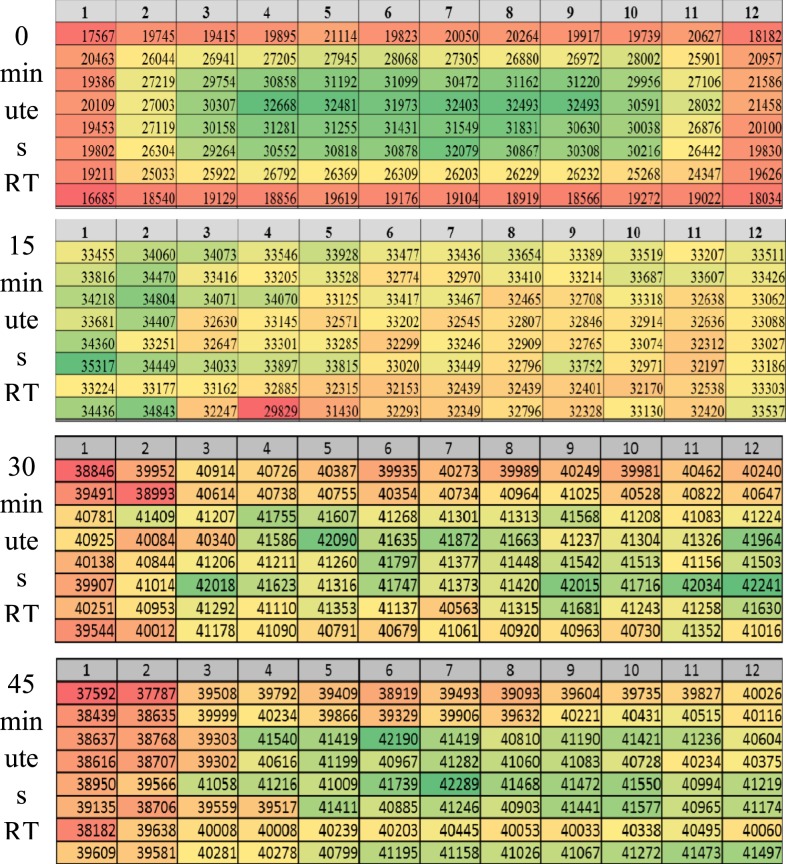
DRAQ5 staining counts and heat map for plates seeded with and without incubation at room temperature (RT) before overnight incubation at 37°C.

**Figure 6.**
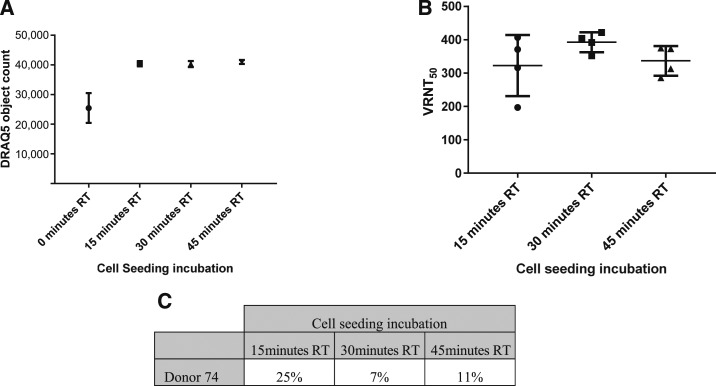
DRAQ5 object count for plates not held or held at room temperature before placing at 37°C overnight (**A**) and corresponding Virus reduction neutralization test (VRNT_50_) titers for selected 30 minutes time point and gated (15, 45 minutes) times (**B**) and % coefficient of variation (**C**).

For correlation testing in the VRNT, the number of samples per plate and plates per batch (maximum number of plates per analysts) were increased to mimic a clinical testing environment. This testing revealed a higher than expected retest rate (8–28%) due to replicate ratio failure (> 2-fold) among the four DEN viruses, mainly from samples located at the top and bottom of the plate. After transfer of the assay to Q2 Solutions Vaccines, an even larger replication ratio failure rate was observed again from samples located at the top and bottom of the plate. Subsequent experiments with DRAQ5 indicated that there was a marked difference in the number of cells around the perimeter of the plate versus the plate interior (Figure 5). It was determined that this phenomenon was also evident before the start of the assay and occurred at the cell seeding step. It was hypothesized that cell attachment was inhibited in the perimeter of the plate due to the rapid heating at 37°C of the wells exposed to the exterior versus a potential insulation effect of the interior wells. To overcome this effect, cells were seeded and plates were held at RT before placing in the 37°C incubator overnight. The result was an elimination of the edge effect with as little as 15 minutes at RT before placing the plates in the 37°C incubator. To confirm the loss of edge effect and improved consistency of replicate titers, each of the three plates were seeded with 30,000 cells, with one plate kept at RT for either 15, 30, or 45 minutes before being placed at 37°C overnight. These times were chosen as 30 minutes was considered an optimal time for analysts and 15/45 minutes gated around this preferred time (Figure 6A and B). The same test sample was evaluated across the three plates. In each case, the sample passed with replicate ratios close to 1.0, indicating that the elimination of the edge effect improved the consistency of the replicate titers. The % CV in this experiment ranged from 7% to 25%, and 30,000 cells per well kept at RT for 30 minutes were selected as the optimal condition (Figure 6C).

The VRNT counts individually infected cells rather than large overlapping foci (FRNT), and unlike traditional microneutralization assays, which rely on a whole well intensity or reporter viruses,^17–21^ the VRNT is rapid, counting virus-infected cells 1 day postinfection and uses unaltered wild-type virus. Compared with other microneutralization assays, including the FRNT adapted to the 96-well format, which counts spots > 2 days, the VRNT offers additional advantages in the ability to count one infected cell within 1 day, rather than waiting for overlapping foci to form, which ensures accuracy and contributes to the increased precision and sensitivity of the VRNT. The VRNT considerably reduces labor and analysis time, eliminates manual plaque counting and significantly increases throughput. This novel neutralization platform correlates well with FRNT and is an alternative to the gold standard FRNT for DEN virus vaccine candidates. Although not presented here, experiments with viruses from unrelated families show that the VRNT is a universal platform and can be widely used.

## Supplementary Material

Supplemental table and figure

## References

[1] WHO, 2017 Dengue Fact Sheet. Geneva, Switzerland: World Health Organization.

[2] Green S Rothman A, 2006 Immunopathological mechanisms in dengue and dengue hemorrhagic fever. Curr Opin Infect Dis 19: 429–36. 16940865 10.1097/01.qco.0000244047.31135.fa

[3] WHO, 2013 Guidelines on the Quality, Safety and Efficacy of Dengue Tetravalent Vaccines (live, attenuated) Technical Report Series. Geneva, Switzerland: World Health Organization.

[4] Hadinegoro SR 2015 Efficacy and long-term safety of a dengue vaccine in regions of endemic disease. N Engl J Med 373: 1195–206. 26214039 10.1056/NEJMoa1506223

[5] Godoi IP 2017 CYD-TDV dengue vaccine: systematic review and meta-analysis of efficacy, immunogenicity and safety. J Comp Eff Res 6: 165–180. 28084784 10.2217/cer-2016-0045

[6] WHO, 2012 Global Strategy for Dengue Prevention and Control, 2012–2020. Geneva, Switzerland: World Health Organization Press.

[7] Durbin AP 2001 Attenuation and immunogenicity in humans of a live dengue virus type-4 vaccine candidate with a 30 nucleotide deletion in its 3′-untranslated region. Am J Trop Med Hyg 65: 405–13. 11716091 10.4269/ajtmh.2001.65.405

[8] Timiryasova TM Bonaparte MI Luo P Zedar R Hu BT Hildreth SW, 2013 Optimization and validation of a plaque reduction neutralization test for the detection of neutralizing antibodies to four serotypes of dengue virus used in support of dengue vaccine development. Am J Trop Med Hyg 88: 962–70. 23458954 10.4269/ajtmh.12-0461 PMC3752766

[9] Torresi J Ebert G Pellegrini M, 2017 Vaccines licensed and in clinical trials for the prevention of dengue. Hum Vaccin Immunother 13: 1059–1072. 28281864 10.1080/21645515.2016.1261770 PMC5443395

[10] Saez-Llorens X. Tricou V Yu D Rivera L Tuboi S Garbes P Borkowski A Wallace D, 2017 Safety and immunogenicity of one versus two doses of Takeda’s tetravalent dengue vaccine in children in Asia and Latin America: interim results from a phase 2, randomised, placebo-controlled study. Lancet Infect Dis 17: 615–625. 28365225 10.1016/S1473-3099(17)30166-4

[11] L'Azou M 2016 Symptomatic dengue in children in 10 Asian and Latin American countries. N Engl J Med 374: 1155–1166. 27007959 10.1056/NEJMoa1503877

[12] Roehrig JT Hombach J Barrett AD, 2008 Guidelines for plaque-reduction neutralization testing of human antibodies to dengue viruses. Viral Immunol 21: 123–32. 18476771 10.1089/vim.2008.0007

[13] Tan C Iglewicz B, 1999 Measurement-methods comparisons and linear statistical relationship. Technometrics 41: 192–201.

[14] Lin LI, 1989 A concordance correlation coefficient to evaluate reproducibility. Biometrics 45: 255–68. 2720055

[15] Salje H Rodríguez-Barraquer I Rainwater-Lovett K Nisalak A Thaisomboonsuk B Thomas SJ Fernandez S Jarman RG Yoon IK Cummings DA, 2014 Variability in dengue titer estimates from plaque reduction neutralization tests poses a challenge to epidemiological studies and vaccine development. PLoS Negl Trop Dis 8: e2952. 24967885 10.1371/journal.pntd.0002952 PMC4072537

[16] Rainwater-Lovett K Rodriguez-Barraquer I Cummings DA Lessler J, 2012 Variation in dengue virus plaque reduction neutralization testing: systematic review and pooled analysis. BMC Infect Dis 12: 233. 23020074 10.1186/1471-2334-12-233 PMC3519720

[17] Shan C Xie X Ren P Loeffelholz MJ Yang Y Furuya A Dupuis AP 2nd Kramer LD Wong SJ Shi PY, 2017 A rapid Zika diagnostic assay to measure neutralizing antibodies in patients. EBioMedicine 17: 157–162. 28283425 10.1016/j.ebiom.2017.03.006 PMC5360589

[18] Song KY 2014 A novel reporter system for neutralizing and enhancing antibody assay against dengue virus. BMC Microbiol 14: 44. 24548533 10.1186/1471-2180-14-44 PMC3930823

[19] Yang H Baker SF González ME Topham DJ Martínez-Sobrido L Zand M Holden-Wiltse J Wu H, 2016 An improved method for estimating antibody titers in microneutralization assay using green fluorescent protein. J Biopharm Stat 26: 409–420. 26010892 10.1080/10543406.2015.1052475 PMC4661136

[20] Maistriau M Carletti T Zakaria MK Braga L Faoro V Vasileiadis V Marcello A, 2016 A method for the detection of virus infectivity in single cells and real time: towards an automated fluorescence neutralization test. Virus Res 237: 1–6. 10.1016/j.virusres.2017.05.004 28501626

[21] Lin R Heeke D Liu H Rao E Marshall JD Chio V Cataniag F Yu L Zuo F McCarthy MP, 2017 Development of a robust, higher throughput green fluorescent protein (GFP)-based epstein-barr virus (EBV) micro-neutralization assay. J Virol Methods 247: 15–21. 28457783 10.1016/j.jviromet.2017.04.012

