# Supplementary material for: Virus Reduction Neutralization Test: A Single-Cell Imaging High-Throughput Virus Neutralization Assay for Dengue
Source: Am J Trop Med Hyg. 2018 Oct 22;99(6):1430–9. doi: 10.4269/ajtmh.17-0948 (PMC6283513; doi:10.4269/ajtmh.17-0948)
Supplement: Supplementary file 1 [file tpmd170948.SD1.pdf]

Supplementary Figure 1. DEN2 assay positive control tracking. Dotted lines represent the mean $VRNT_{50}$ (middle line) and ± two standard deviations about the mean (upper and lower lines).

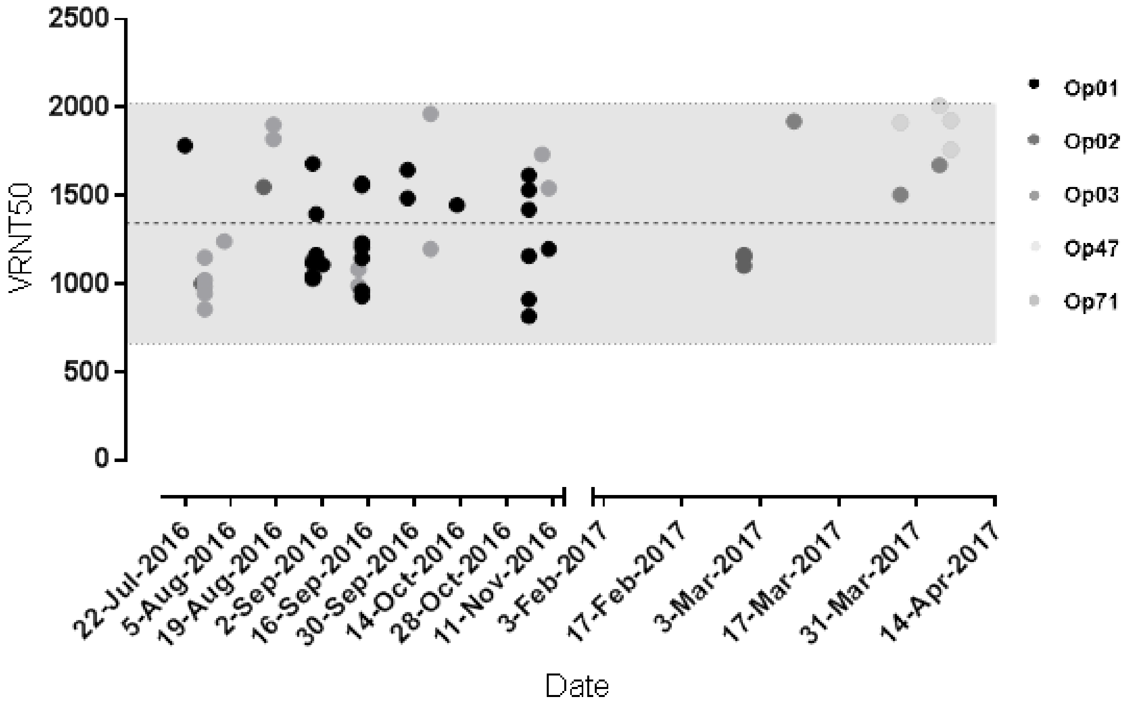

Supplementary Table 1. FRNT50 titers and corresponding titer fold-change from Day 1 titers.

	FRNT50 titer			
Day	DEN1	DEN2	DEN3	DEN4
1	247	1264	617	148
2	64	417	730	90
3	69	235	255	68
4	164	38	192	18

	Titer Fold-change			
Day	DEN1	DEN2	DEN3	DEN4
1	n/a	n/a	n/a	n/a
2	3.9	3	1.2	1.7
3	3.6	5.4	2.4	2.2
4	1.5	33.3	3.2	8.2

Supplementary Table 2 FRNT$_{50}$ and VRNT$_{50}$ correlation titers for DEN1-4

	DEN1		DEN2		DEN3		DEN4	
Sample	VRNT50	FRNT50	VRNT50	FRNT50	VRNT50	FRNT50	VRNT50	FRNT50
1	<10	<10	<10	<10	<10	<10	<10	<10
2	<10	<10	<10	<10	28	<10	<10	<10
3	<10	<10	<10	<10	<10	<10	<10	<10
4	<10	<10	16	26	<10	<10	<10	<10
5	10	<10	<10	<10	73	37	<10	<10
6	<10	<10	<10	<10	<10	<10	<10	<10
7	<10	<10	<10	<10	<10	<10	<10	<10
8	<10	<10	<10	<10	<10	<10	<10	<10
9	21	14	11	<10	91	68	<10	<10
10	226	123	109	326	380	679	53	<10
11	312	285	105	106	282	714	156	55
12	<10	<10	<10	<10	<10	<10	<10	<10
13	<10	<10	<10	<10	<10	<10	<10	<10
14	25	<10	12	<10	73	39	<10	<10
15	151	131	99	141	697	1309	92	17
16	484	689	255	235	1101	2007	541	153
17	530	176	225	606	492	1549	530	91
18	<10	<10	<10	<10	29	<10	<10	<10
19	228	193	153	282	637	4192	112	164
20	105	71	43	70	293	412	69	17
21	850	459	240	342	660	2860	850	69
22	325	64	88	181	487	406	184	28
23	<10	<10	<10	<10	<10	<10	<10	<10
24	<10	<10	<10	<10	<10	<10	<10	<10
25	501	370	265	580	1492	1557	975	260
26	424	154	899	824	611	1045	769	291
27	<10	<10	11	<10	<10	<10	<10	<10
28	400	311	639	833	1028	1168	378	79
29	<10	<10	<10	<10	<10	<10	<10	<10
30	<10	<10	<10	<10	<10	<10	<10	<10
31	51	<10	34	40	316	464	<10	<10
32	<10	<10	<10	<10	<10	<10	<10	<10
33	<10	<10	<10	<10	<10	<10	<10	<10
34	<10	<10	<10	<10	<10	<10	<10	<10
35	969	495	713	447	>5120	4368	291	52
36	<10	<10	<10	<10	<10	<10	<10	<10
37	<10	<10	<10	<10	<10	<10	<10	<10
38	<10	<10	<10	<10	<10	<10	<10	<10
39	1479	259	1379	825	3062	4324	541	101
40	<10	<10	<10	<10	<10	<10	<10	<10
41	95	13	24	68	<10	45	31	23
42	<10	<10	<10	<10	<10	<10	<10	<10
43	<10	<10	<10	<10	<10	<10	<10	<10
44	<10	<10	<10	<10	<10	<10	<10	<10
45	<10	<10	<10	<10	<10	<10	<10	<10
46	<10	<10	<10	<10	<10	<10	<10	<10
47	<10	<10	<10	<10	<10	<10	<10	<10
48	17	<10	12	<10	38	16	<10	<10
49	<10	<10	<10	<10	<10	<10	<10	<10
50	<10	<10	<10	<10	<10	<10	<10	<10
51	16	<10	<10	<10	21	<10	<10	<10
52	<10	<10	<10	<10	26	<10	<10	<10
53	64	33	22	20	63	46	29	<10
54	34	<10	29	22	31	29	18	<10
55	<10	<10	<10	<10	<10	<10	<10	<10
56	117	50	164	379	584	277	76	26
57	61	49	46	58	162	87	26	<10
58	<10	41	34	35	142	96	37	23
59	<10	<10	<10	<10	12	<10	<10	<10
60	292	88	784	1488	822	638	288	55
61	<10	<10	<10	<10	<10	<10	<10	<10
62	<10	44	17	10	237	43	167	45
63	33	20	190	187	29	28	17	10
64	<10	64	64	48	268	135	70	36
65	50	<10	<10	<10	<10	<10	<10	<10
66	<10	<10	<10	<10	89	52	<10	<10
67	379	333	188	413	1017	1515	66	54
68	168	57	53	52	460	337	41	<10
69	163	<10	<10	<10	18	<10	<10	<10
70	<10	<10	<10	<10	<10	<10	<10	<10
71	<10	<10	<10	<10	<10	<10	<10	<10
72	551	290	204	418	1464	5214	483	136
73	17	<10	11	<10	52	<10	<10	<10
74	<10	<10	<10	<10	<10	<10	<10	<10
75	45	39	297	252	102	159	15	<10
76	36	41	31	37	321	525	64	15
77	<10	<10	<10	<10	<10	<10	<10	<10
78	20	25	19	14	89	109	13	<10
79	1015	172	223	409	1272	906	190	143
80	150	75	225	391	570	663	285	190
81	59	49	128	65	129	96	68	<10
